# Multimodal Human and Environmental Sensing for Longitudinal Behavioral Studies in Naturalistic Settings: Framework for Sensor Selection, Deployment, and Management

**DOI:** 10.2196/12832

**Published:** 2019-08-20

**Authors:** Brandon M Booth, Karel Mundnich, Tiantian Feng, Amrutha Nadarajan, Tiago H Falk, Jennifer L Villatte, Emilio Ferrara, Shrikanth Narayanan

**Affiliations:** 1 Signal Analysis and Interpretation Laboratory University of Southern California Los Angeles, CA United States; 2 Multimedia/Multimodal Signal Analysis and Enhancement Lab Institut National de la Recherche Scientifique University of Québec Montréal, QC Canada; 3 Department of Psychiatry and Behavioral Sciences University of Washington School of Medicine Seattle, WA United States; 4 Information Sciences Institute University of Southern California Marina del Rey, CA United States

**Keywords:** research design, human activities, behavioral research, longitudinal studies, wearable electronic devices, organizational case studies, in situ research

## Abstract

**Background:**

Recent advances in mobile technologies for sensing human biosignals are empowering researchers to collect real-world data outside of the laboratory, in natural settings where participants can perform their daily activities with minimal disruption. These new sensing opportunities usher a host of challenges and constraints for both researchers and participants.

**Objective:**

This viewpoint paper aims to provide a comprehensive guide to aid research teams in the selection and management of sensors before beginning and while conducting human behavior studies in the wild. The guide aims to help researchers achieve satisfactory participant compliance and minimize the number of unexpected procedural outcomes.

**Methods:**

This paper presents a collection of challenges, consideration criteria, and potential solutions for enabling researchers to select and manage appropriate sensors for their research studies. It explains a general data collection framework suitable for use with modern consumer sensors, enabling researchers to address many of the described challenges. In addition, it provides a description of the criteria affecting sensor selection, management, and integration that researchers should consider before beginning human behavior studies involving sensors. On the basis of a survey conducted in mid-2018, this paper further illustrates an organized snapshot of consumer-grade human sensing technologies that can be used for human behavior research in natural settings.

**Results:**

The research team applied the collection of methods and criteria to a case study aimed at predicting the well-being of nurses and other staff in a hospital. Average daily compliance for sensor usage measured by the presence of data exceeding half the total possible hours each day was about 65%, yielding over 355,000 hours of usable sensor data across 212 participants. A total of 6 notable unexpected events occurred during the data collection period, all of which had minimal impact on the research project.

**Conclusions:**

The satisfactory compliance rates and minimal impact of unexpected events during the case study suggest that the challenges, criteria, methods, and mitigation strategies presented as a guide for researchers are helpful for sensor selection and management in longitudinal human behavior studies in the wild.

## Introduction

### Overview

Recent advances in portable consumer technologies have led to a surge in the development of electronic devices [1] for monitoring and tracking human activity, wellness, and behavior. Aided by the ubiquity of personal smartphones, Bluetooth, and Wi-Fi, many devices currently on the market can discreetly collect physiologic and behavioral signals and upload the information to remote servers. Because of the growing support for distributed and personalized sensing, diverse research communities are taking a keen interest in this field, empowering the coordination of research studies of populations outside the laboratory and in natural home or work environments (also known as studies *in the wild*) [2]. For research into everyday human behavior, such as daily routines, studies conducted in natural settings can yield more relevant and insightful data than those performed in the laboratory [3-8].

Several factors need to be considered for the collection of data in natural human settings using sensing devices. Different sensors have different sampling rates, power restrictions, and communication capabilities. Participants also have their own habits and daily routines into which the sensors and the data collection procedures need to be embedded. A data collection framework designed to operate in the wild should therefore be flexible enough to accommodate different data communication channels and be capable of capturing information from different people with different needs at different times. These factors and a host of other challenges mentioned in this work complicate the data collection process and ultimately affect the quality of data available for analysis.

### Background

Several previous studies have described some of these challenges [9-11] and offered strategies for mitigating them [12-15]. Other works offer data collection plans for particular fields of study that address the unique concerns of their research areas [16,17]. This paper subsumes many of the challenges and suggestions from these other works and aims to provide a comprehensive collection of methods and suggestions that help researchers address the challenges related to sensor selection and management in research studies. It specifically focuses on longitudinal studies aiming to unobtrusively capture and assess aspects of human experience and natural behavior; thus, it assumes a participatory study framework instead of a provocative approach [2]. Some examples of unobtrusive human behavior studies are *StudentLife* [6], *AffectiveROAD* [18], and a dataset on emotion recognition from wearable physiological sensing [19].

### Objectives

**Figure 1:**
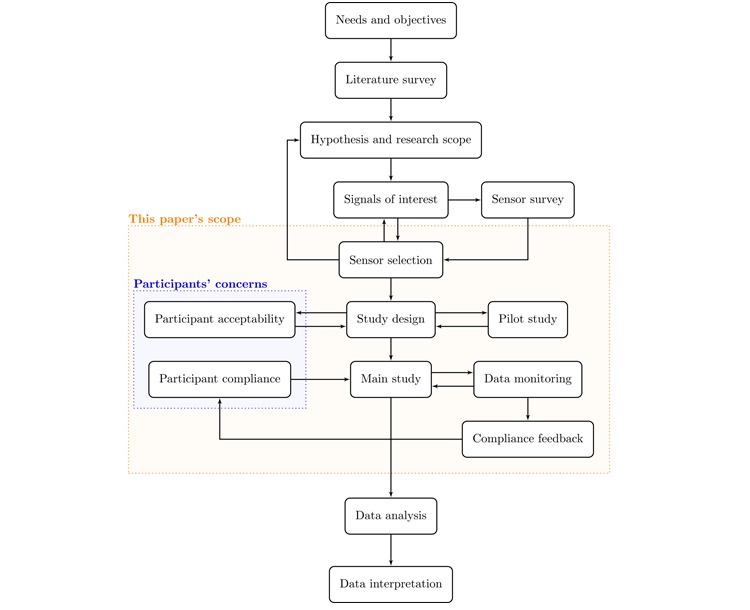
An overview of the general scientific process for human research studies involving sensing.

Figure 1 illustrates a sequence of research program states at various stages for these types of studies. The scope of this paper covers the preplanning stages pertaining to sensor selection and the stages during a study related to sensor and data management. The key assumptions were as follows: (1) researchers already have a clear research objective in mind and have researched previous literature to develop a sense of the types of physiological and behavioral signals that may be helpful in achieving the goals and (2) researchers have surveyed the landscape of sensing technologies and are beginning to design a study protocol and select the appropriate sensors.

To the best of the authors’ knowledge, this paper represents a first attempt to present a comprehensive guide for selecting and managing sensors for *in situ* research studies. The guide is based on a survey of related work [9-15] and the authors’ experiences in designing a multiweek research study. It describes the main challenges that differentiate longitudinal and unobtrusive [20] studies in the wild from studies conducted in controlled laboratory settings. It also provides an overview of modern portable sensing capabilities and information workflows and outlines a general data collection framework that leverages an internet-enabled infrastructure for real-time data collection and feedback. It enumerates several criteria (or dimensions) that researchers should consider when designing a data collection protocol using portable sensors for a known participant population, and discusses the manner in which these dimensions can affect human subjects’ concerns and data quality. Furthermore, it illustrates a snapshot of some of the many consumer technologies and products available for sensing in the wild as of mid-2018. This case study employs all the criteria and methods discussed, evaluating these with respect to participant compliance and the number of notable unplanned events occurring during data collection.

## Methods

### Overview

This section discusses the challenges involved in designing protocols and using sensors to collect data about human behavior in the wild, presenting a general-purpose framework that researchers can use for envisioning and orchestrating sensor data flow. This framework subsumes the most common information flows provided by modern sensing technologies. This section also presents an exposition of the various criteria and dimensions for which all sensors should be evaluated before the beginning of the data collection period, as well as a snapshot summary of many modern consumer technologies and products available for each type of sensing. The checklist form in Multimedia Appendix 1 provides a concise checklist of the challenges in this section. The authors’ hope is that researchers will use this checklist in their discussions and planning about protocol design to help account for the numerous sensing challenges.

### Challenges and Risk Mitigation Strategies

Studies conducted outside of controlled laboratory settings are of interest to researchers, as participants can be examined in their day-to-day environments where natural behaviors occur. Nevertheless, in the wild, many potentially confounding variables cannot be fully controlled, yielding unpredictable sources of variability alongside logistical difficulties. Some challenges in this kind of data collection are mitigated through careful planning and effective communication before the study begins. Other challenges are predictable, but they occur spontaneously, and they must be managed reactively with the aid of semiautomated systems. This section highlights the primary difficulties that are unique to studies in the wild and suggests strategies to help overcome them.

#### Sensor Logistics, Deployment, and Maintenance

One of the foremost difficulties is the logistical burden of deploying and maintaining sensors. As research teams have limited direct control over the environment for *in situ* studies, they should be aware of the different potential sensor failure modes and have a plan for quickly detecting and recovering from them.

Sensor failure is often inevitable, especially for studies conducted at scale, and an effective solution is to simply replace the devices by preplanning to streamline this process. For example, arranging to have trained personnel available to meet with participants in their environment to swap defective devices can minimize data losses because of downtime. For sensors deployed in the environment itself (as opposed to wearable sensors), devising a mounting scheme that will allow for easy replacement may also help.

To aid the tracking of the status of all sensors in a large study, planning in an upfront manner to create semiautomated tools that monitor the state of each sensor as often as possible can help identify failures quickly, report them to personnel for maintenance, and further decrease data collection downtime [21]. Moreover, the use of automated tools may become a necessity if the number of participants, sensors, or hours of recording becomes large. Data-driven approaches for detecting and identifying anomalous sensor data streams have been recently proposed in the literature [22,23]. Implementing a strategy for automated ongoing maintenance of the deployed sensors is much easier once the research team has direct access to recent sensor data. A data flow framework (presented in a later section; *Information Flow Layers*) outlines and describes the communication channels that carry sensor data to the data servers (the collection of systems where the data are collected and securely stored for later processing). Researchers can use this framework to plan communication paths for each sensor and then set up a script to run on the research server, which monitors these data streams and notifies assistants when sensors malfunction. For example, automatic programs can be used to assess the quality of electrocardiography (ECG) signals and give feedback to the research support staff about potential fitting and usage problems [24].

Specific logistics and deployment strategies will be unique to each research study, and they will largely be influenced by the restrictions and constraints imposed by the research environment. For example, some hospitals require all equipment to be powered using 3-prong plugs; therefore, all sensor chargers are required to be used through 3-prong adapters. Other restrictions may include Wi-Fi availability, permission to mount sensors on the walls, availability of charging ports for sensors, and space for sensor storage, to name a few. Permissions for the research personnel to access all areas in which the study takes place should also be considered.

#### Data Loss

Data loss may occur for several reasons, including sensor or data pipeline malfunctions, poor participant compliance, and attrition, among other reasons. For example, sensors may fail to deliver data, as they run out of battery power or break, or they may fail to deliver when network outages interfere with data transfers. Subjects may also neglect the data collection protocol (including forgetting to wear the sensor or wearing the sensors without following instructions), forget to recharge a worn device, or fail to upload data at the end of each session, for example, the *Hexoskin* garment requires manual data upload via Universal Serial Bus (USB). In more extreme cases, subjects may become frustrated with the study and elect to drop out, thereby reducing the total amount of available data.

The key to mitigating these various sources of data loss is being aware of where in the data stream pipeline the losses occur. The *Data Acquisition and Flow Framework* section of this paper enumerates the communication paths that help carry sensor data to their destination on a research server. Once researchers have decided on a sensor suite, and once they know which paths are required, small scripts or monitoring systems can be instrumented to test or infer status of each communication channel and report failures to the research team. For cases where data loss occurs at the source (ie, the participants), this section describes a mechanism for sending feedback to the participants to notify them of the data loss and encourage them to remedy it.

#### Data Signal Quality and Unintentional Variability

Related to data loss, the signal quality of sensor data is a concern that presents a substantial challenge for research in the wild. The term *signal quality* used here refers to the ability of each sensor to measure its signal(s) of interest. Poor data quality may occur when sensors are not properly worn or maintained, such as when a wristband photoplethysmography (PPG) sensor to measure heart rate is worn too loosely or when a microphone is obscured. Instances of improper or inconsistent sensor usage are inevitable in large studies in the wild, and they can lead to an unintentionally higher degree of variability in the data captured across all participants, which may consequently skew the resulting statistical analyses.

Early steps should be taken to ensure that participants receive proper training for using the adopted sensors before the study begins and that clear and accessible instructions are made available to serve as a reminder. Making plans to monitor the quality of sensor data streams so that appropriate actions can be taken to rectify problems is also highly beneficial, especially for long-term studies. Once a process is in place to determine the quality rating of recent data, different intervening actions may be appropriate, depending on the participant population, study environment, and the goals of the research project. Some example interventions for improving data quality include the following: retraining participants in sensor usage, adjusting sensor fit, improving the network infrastructure to reduce downtime, or simply sending reminders to participants (eg, smartphone push notifications) to remind them to wear their devices and upload the data. Quickly responding to rectify data quality drops can help preserve the value of the data and minimize data loss. If low-quality data persist despite these measures, automated signal enhancement methods may still be employed to algorithmically improve data quality. The *Data Acquisition and Flow Framework* section illustrates how data from the sensors can be aggregated on a research server.

#### Privacy and Security

Among the opportunities to generate scientific knowledge are significant challenges to the ethical conduct of research on human subjects [25]. Threats to privacy and data security constitute the greatest risk to participants of behavioral research in the wild. As sensing technologies become ubiquitous and data science advances, it is possible to use passively collected digital data to identify and predict a surprising range of human behaviors with increasing accuracy [26]. Participants are often unaware that when they consent to share data from their fitness tracker, they may be allowing researchers to infer information about their alcohol consumption, sexual activity, and mental health symptoms. The accidental or malicious release of this information could cause significant social, occupational, and psychological consequences to participants. The informed consent process must provide clear and transparent communication about what data are collected and how the data will be used by researchers, how data are either anonymized or kept confidential, and how data are securely transferred, stored, and destroyed. Researchers must stay up to date on evolving privacy and security concerns and best practices for mitigating risk.

Another significant challenge when conducting studies in the wild is respecting and protecting the privacy of nonparticipants coincidentally present in the research environment. For research scenarios in which raw audiovisual data are collected, extra steps must be taken to ensure that either no personally identifiable information (PII) is recorded about nonparticipants or that they are informed that they may be recorded, where appropriate and depending on municipal or state regulations and institutional review board (IRB) approval. A tactic for avoiding the collection of PII, even accidently, is to immediately transform the collected raw data streams, such as audio or video, into anonymized features—intonation, mel-frequency cepstral coefficients, gestures, and posture—and record these instead [27].

Another important step toward maintaining privacy is to ensure secure transmission of sensor data to the research server with as few transfers to intermediate nodes as possible. The *Criteria Related to Protection of Human Subjects* section in this paper discusses methods for securely transmitting information across a network, and the *Data Acquisition and Flow Framework* section can help researchers plan secure communication paths.

### Data Acquisition and Flow Framework

State-of-the-art electronics and sensing technologies offer a wide variety of communication protocols for sending information among devices. Selection of the appropriate sensors for a research project depends on many factors, which are discussed in more detail in the *Considerations and Criteria for Sensor Selection* section. A crucial step toward evaluating each sensor is to understand the ways in which its data can be transmitted through different communication channels and how its data flow may be affected by the choice of other sensors and data hubs.

The proposed general sensing framework, deemed suitable for studies in the wild, depicts common transmission paths for data flowing from multiple sensor streams through disparate network paths and arriving on a secure server that is accessible only by the research team. The framework aggregates data in a single place, allowing for simpler implementations of automatic stream monitoring and participant feedback systems.

#### Information Flow Layers

**Figure 2:**
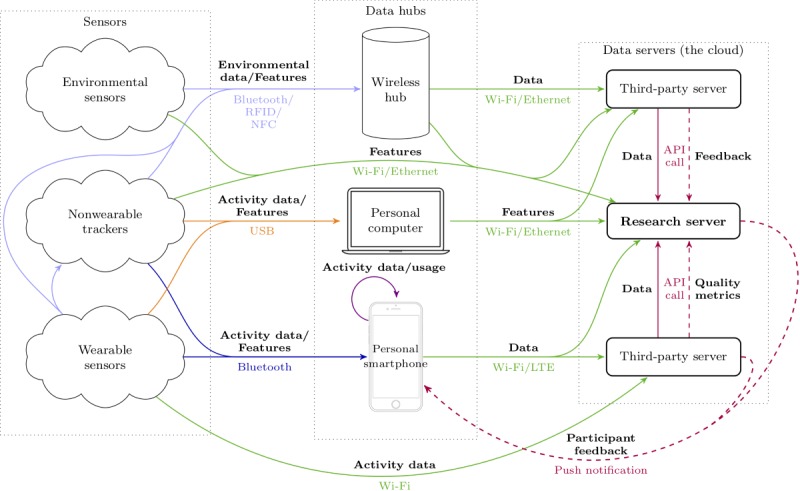
A framework for studies of human behavior in the wild, showing common potential information pathways for data produced by sensors (eg, physiologic and activity), destined to be stored on a single research server. This type of data flow paradigm enables centralized data monitoring and facilitates immediate automatic participant feedback regarding data quality and compliance via the participant’s smartphones. RFID: radio-frequency identification; NFC: near-field communication; USB: universal serial bus; API: application programming interface.

Figure 2 depicts potential information pathways through different communication channels for passing data obtained from sensors (in the left column) to data servers (right column), where all the information passes through an intermediate data hub layer (middle column). These intermediate hubs are any devices that act as bridges to facilitate the aggregation and delivery of transient sensor data into long-term storage. Most of the available sensors in the market support a data flow matching some combination of paths in this figure.

The primary aim in this framework is the aggregation of all sensor data onto a single research server where additional processing, monitoring, and feedback can be performed. The following subsections describe each of these layers (columns) in detail.

#### Sensors

Sensors for studies in the wild can broadly be grouped into 3 categories: environmental sensors, nonwearable (human) trackers, and wearable sensors.

##### Environmental Sensors

These devices passively capture information about their surroundings. Some examples of data captured by these types of sensors include the following: temperature/humidity/CO_2_ levels, inertial measurements (eg, from accelerometers, gyroscopes, or magnetometers), and acoustics. These devices often perpetually broadcast information about their surroundings, using low-energy Bluetooth or radio-frequency identification (RFID) signals. Sampling rates below 1 Hz or event-based sampling techniques are typical, as environmental data usually change slowly (at least compared with physiological signals).

##### Nonwearable Trackers

These devices are placed in the environment, and they capture information about subjects and their behaviors indirectly or in a passive way. A few instances of these types of devices include the following: RFID scanners, Doppler effect and under-the-mattress sleep trackers, infrared gaze trackers, and video cameras. These sensors often operate on wall power and may include network capabilities for simplifying data transmission to long-term storage on the data servers. They also often include companion websites or smartphone apps for visualizing metrics extracted from the sensor data.

##### Wearable Sensors

These types of sensors encompass the set of custom-built or consumer products that are worn or carried on the subject’s body to collect physiologic or contextual data or features extracted from data, for example, heart rate from electrocardiogram data, for behavior and activity tracking. Devices such as smart watches/wristbands, smart undergarments (underwear, T-shirts, and bras that collect data), smart rings, voice activity detectors, and smart shoe soles are some examples. Many of these devices can be recharged for long-term use over multiple sessions, and they generally either communicate via Bluetooth with companion apps installed on users’ smartphones or via USB connections with personal computers. The companion apps tend to provide visualizations of the received data and upload functionality for long-term storage on third-party servers. Interestingly, when running certain tracking apps in the background, smartphones themselves can also serve as wearable sensors, collecting information about user movement and smartphone usage patterns.

#### Data Hubs

Data hubs are devices dedicated to collecting, aggregating, and transmitting sensor information to data servers. Transient data sources, such as many environmental sensors, have little memory, and they need to have their data collected continuously and retransmitted to a data server for long-term storage. Wi-Fi–enabled data hub devices with Bluetooth capabilities can serve as conduits for these types of data streams, whereas personal computers can act as data hubs for USB-only sensors. Battery-powered sensors collecting data at a high rate usually communicate via USB, as the bandwidth and transmission speed are higher, and as wireless data transmission drains more power. Battery-powered sensors can afford to send a smaller amount of data through Bluetooth, whereas smartphones can often serve as both data hubs and data visualizers.

#### Data Servers

The term *data servers* refers to the collection of machines in which all the sensor data are stored. A typical consumer off-the-shelf sensing device will provide some pipeline for getting data off the sensor and into a data store in the cloud, usually owned by the sensor product’s company. These companies often provide an application programming interface (API) for accessing the data, using automated tools that transmit the data securely to protect subject privacy. Eventually, all the sensor information needs to be aggregated in a single place, the research team’s own server, so that the team has permanent and easy access to it. The aggregation of all sensor data on this server continuously throughout the data collection process enables monitoring and feedback systems to help manage some of the challenges mentioned in the *Studies in the Wild: Challenges and Risk Mitigation Strategies* section.

### Considerations and Criteria for Sensor Selection

Minimizing participant risk and burden while maximizing the amount and quality of data is of primary importance. The set of sensors used plays a major role in a study’s outcome, as data quality is inherently constrained by the sensors’ characteristics and the participants’ interactions with those sensors. Selecting the appropriate sensors to employ in a research study can be complicated, as the market provides many options, and each device has unique qualities and capabilities.

This section establishes a comprehensive list of the different criteria that should be considered before data collection begins. In practice, researchers must strike a balance between meeting their research objectives and ensuring a smooth participant experience to maximize attrition and minimize data loss. Both needs are constrained by the properties of the sensors that are available or can be produced. The criteria are partitioned according to whether each criterion is a characteristic of the sensor or whether it primarily concerns either the researchers or the participants. It is important for researchers to carefully review each one of these criteria, as they are highly connected, and each choice affects the outcome and experiences for both researchers and participants.

Table 1 lists different sensor criteria grouped according to whether they primarily concern research objectives and logistics, sensor characteristics, participant engagement, or human subject protection during the study. The categorization is not perfect, as some of the criteria pertain to more than 1 group, but it helps emphasize the different perspectives researchers should examine when selecting sensors. Key criteria are included in this table, which are expected to remain relevant as technology changes; however, there may be other factors worth considering, depending on the specific needs of a research project. The following subsections describe each criterion in detail.

**Table 1:** Considerations and criteria for sensor selection.

Research objectives and logistics	Sensor characteristics	Participant engagement	Human subject protection
Signals of interest	Sensor customizability	Cohort and individual suitability	Access and usability
Data properties and quality	Cost	Burden to participants	Privacy
Data access logistics	Battery life	—^a^	Data security
Sensor synergy	Operating system support	—	—
Additional experiment setup costs	Robustness	—	—
Sensor acceptance among target population	Provider support	—	—
On-site infrastructure requirements	—	—	—

^a^Empty cells are filled with a dash for visual clarity.

#### Criteria Related to Research Objectives and Logistics

The criteria in this section pertain to the logistical implications of the selected sensors and ways in which the selection affects the final outcomes and goals of a research project.

##### Signals of Interest

These criteria relate to the signals and how they are measured.

###### Target Signals

Before data collection begins, researchers need to consider what type of signals they want to measure from the participants or the environment. Varying amounts of potentially relevant information can be obtained from signals collected from different sources, such as physiology, for example, heart rate, breathing rate, electrodermal activity (EDA), behavior (eg, time spent speaking, sleeping duration and stage progression, number of steps per time interval, social interactions, and surveys), and the environment (eg, temperature, humidity, and CO_2_ levels). The utility and overall quality of the chosen signals depend on the sensors’ measurement mechanisms.

###### Measurement Mechanism

The physical mechanism through which a signal is acquired affects its quality and overall utility for future analysis. As an example, human location and kinematic data can either be reconstructed from a series of Global Positioning System (GPS) coordinates or inferred from an inertial measurement unit (IMU), such as an accelerometer and gyroscope. The GPS data tend to produce more accurate location measurements and less accurate kinematic ones; the IMU location accuracy drifts over time but yields better kinematic figures, whereas GPS data can be used in the aid of calibrating step count from an IMU [28]. Another example is heart rate data, which can be obtained through PPG or ECG, each of which yields significantly different signal qualities and properties. The measurement mechanism may be constrained by a sensor’s form factor requirements (wristband vs garment), which may limit the quality of data that can be obtained.

##### Data Properties and Quality

These criteria are important for assessing the quality and potentially undesirable aspects of gathered data.

###### Sampling Rate

For most consumer sensing technologies, the sampling rate is fixed by hardware design and power constraints, and it cannot be altered. It is always possible to decrease the number of samples considered for analysis purposes by downsampling data originally collected at a higher rate. However, upsampling data collected at a lower rate introduces distortions into the signal [29], and that may impact its utility for later analysis. The sampling rate of any sensing device should be at least twice that of the desired underlying signal’s maximum frequency for the recording to provide reasonable fidelity (per the Nyquist-Shannon sampling theorem). The human voice, for example, can be characterized by pitch and formants (among many other features), which require sampling rates at least twice the maximum vocal frequency (typically greater than 8 kHz) for adequate analysis. However, tracking the position of a person inside of a building can be sampled around once per second, with meter-level accuracy based on average indoor walking speeds [30]. Researchers should be aware of the analytical power of the target signals and choose sensors capable of capturing data at a frequency where meaningful information can be extracted.

###### Signal-to-Noise Ratio

The data will only be useful if the signal-to-noise ratio (SNR) of the measurements is higher than a certain threshold. Noise in this case refers to any unwanted alterations to a signal during the measurement process, and it can appear for many reasons. If the noise is too high, it might not be possible to extract the relevant information from the measurements. For example, ECG-based heart pulse measurements may be subjected to noise when a participant moves or when the electrodes attached to the skin briefly detach during physical activity. Audio recordings of people socializing may also include unwanted background sounds. As unexpected sources of noise can occur in a research study, test runs with a small cohort should be conducted for sensors under consideration and then inspected to determine whether the SNR is adequate to extract meaningful information. Researchers may be able to improve a sensor’s SNR by understanding where noise is introduced into the measurements and taking steps to reduce it.

###### Accuracy and Precision

Accuracy refers to the bias of the measurements, and precision is a representation of the variance of the measurements over time. High-accuracy (low bias) and high-precision (low variance) sensors are the most desirable. Published scientific validation studies pertaining to the accuracy and precision of measurements are available for some commercial and research-grade sensors. In situations where no previous validation work exists for a device, researchers should consider performing their own validation tests, using state-of-the-art, gold-standard sensors as the basis for comparison. As an example, in a study examining the measurement accuracy and precision of wrist-worn PPG devices (eg, Fitbit) among a diverse group of participants performing various physical activities, heart rate measurements were accurate to within 5% of clinical-grade devices, and the measured number of step counts varied within 15% of the actual number [31].

###### Drift

Measurement drift is a natural phenomenon that can occur in any sensor, caused by unintentional modifications to the device or object being measured [32]. When all other factors are held constant, measurements of a signal may drift up or down because of, for example, temperature or humidity shifts, changes in electrode impedance, or physical movement of the body. In many cases, drift is caused by physiological or environmental factors that cannot be controlled in the wild, but there are many common techniques for removing drift effects, including high-pass filters [33], adaptive filters [34], and time-variant filters [35]. In other cases, drift can be caused by sensor wear or material corrosion; therefore, it is important for research teams to consider the impact that normal usage and time will have on the sensors, and it is important to consider how this may cause a drift in the measurements.

###### Data Access at Various Stages of Processing

In some applications, it is important to be able to access the raw (unprocessed and unfiltered) signals. This is most relevant for research involving the denoising of signals, artifact removal, feature extraction, or even the estimation of other data streams from correlated signals [36]. Many consumer sensor devices provide preprocessed signals with artifacts already removed and which have been transformed into higher-level features, such as step count, heart rate, sleep quality, or physical readiness. Some sensor product companies elect to keep their preprocessing techniques unpublished; therefore, it can be difficult for researchers to understand exactly what each feature represents. These ready-made features can be useful for analysis, but researchers should be cautious when using features with no published methodology unless the features have been previously validated in scientific experiments. In cases where a provided feature cannot be trusted or is proven unhelpful in analysis, having access to the raw data to extract more meaningful features may be beneficial.

##### Data Access Logistics

These criteria concern the ease with which data are stored and accessed by researchers.

###### Data Upload Procedure

How and when data are transferred from sensor devices through the network to a data server can have a profound impact on a research project. As far as data upload procedures are concerned, there are 2 types of sensors: the ones that require manual interaction and the ones that automatically and transparently upload data once configured. Manual interaction is often required for devices that collect a large amount of data and need to transfer it in bulk (eg, a Hexoskin ECG sensor uploads to a personal computer via USB). Automatic uploading is typically available for sensors that can stream information transparently to a data hub or smartphone over either Wi-Fi or Bluetooth (eg, an OMsignal ECG sensor uploads data wirelessly to a smartphone app). Both researchers and participants usually benefit from the automated paradigm, as there is less work involved for both parties, and data becomes available sooner, but the researchers need to consider its impact on smartphone battery drain and network bandwidth contention.

###### Ease of Data Access

Once the data have been successfully transferred from sensors to the data servers, data need to be stored on a research server that is easily accessible to the research team. Some sensors may be configured to upload information to the research server directly. For example, some companies supply a website where researchers can log in, visualize, and download participant data. Some companies track uploaded sensor data separately per user, in which case the research team would be responsible for creating and managing the participant accounts. Companies may provide tools to facilitate the download of data, such as Web-based (eg, REST) interfaces or APIs. The existence of well-documented guidebooks or a responsive technical support staff for these tools should be considered when selecting sensors.

##### Sensor Synergy

These criteria concern potential symbioses among sensors and signals.

###### Redundancy of Signals

There are situations in which measuring the same underlying signals using different measurement devices might be advantageous to a research effort. One such circumstance is when a sensor’s accuracy and precision are unknown, but it is otherwise an appropriate pick for research. For example, if this device is a PPG-based wrist-worn sensor for heart rate tracking, then collecting heart information in parallel (perhaps on a subset of the participants), using an ECG sensor that has been validated against a gold standard, can enable researchers to infer the measurement quality of the PPG sensor. In a different scenario, researchers may decide that a certain signal is so important to capture in its full fidelity that using a single sensor that may occasionally fail or experience higher noise levels is not adequate. Using multiple sensors to capture the same signal adds fault tolerance to the measurement of that target signal, and this may also help reduce systemic measurement errors (eg, by averaging).

###### Sensor Versatility

Using a sensor that can adequately serve multiple purposes may be preferable to using multiple sensors instead. There are many reasons why this may be beneficial, such as cost, reductions in participant and research staff burdens, and simplicity. For example, it is possible to program a smartphone to gather human-produced audio and record participant proximity to known locations within a building by exploiting its Bluetooth or Wi-Fi connectivity. This approach uses a single sensor to achieve both goals instead of using 2 separate devices to capture each signal.

##### Additional Setup Costs

These criteria describe the (perhaps hidden) extra time and financial costs associated with setting sensors up for experiments.

###### Installation and Maintenance Costs

Once purchased, sensors require installation and maintenance throughout a research study to ensure measurement consistency and minimize data loss. Some sensors, such as Bluetooth beacons, may come packaged with installation tools that interfere with maintenance objectives (eg, double-stick tape for wall mounting). Using alternative installation devices (eg, adhesive Velcro strips) in anticipation of device malfunctions or required battery replacements can help expedite repairing or replacing these devices when necessary. This may add a small additional per-unit cost to some of the chosen sensors, but this can save time and may help save money in other ways.

###### Participant Training and Support

Participants who will wear sensors throughout a study should be trained to use these devices according to study rules and objectives. Generally, support staffing may be required, as the complexity and number of sensors increases or the sensors’ robustness decreases.

###### Service Costs

Some companies, such as those producing sensors targeted for research rather than consumer use, may offer additional services for some cost. These services may include data aggregation and storage, data visualization, more convenient data access, or real-time monitoring and quality tracking for incoming data. Researchers should identify which services, if any, may be necessary.

##### Sensor Acceptance Among Target Population

Regardless of every desirable quality a sensor may possess for the research team and objectives, it cannot be beneficial if participants recruited from the target population will not accept or use it. There are many reasons why participants may reject any specific sensor, such as discomfort, obtrusiveness, complexity, or fashionability. These objections cannot be anticipated fully; thus, researchers should assess beforehand whether the target population would be generally willing to engage with the potential sensor set selected for use.

##### On-site Infrastructure Requirements

Studies conducted in the wild, which use sensors, depend on the study site infrastructure. As researchers converge on a set of desired sensors for a specific study, the infrastructural resources necessary at the study site(s), which can satisfy the sensor requirements, will emerge. In some cases, the existing infrastructure may not provide the resources required, but it can sometimes be augmented (eg, with additional wireless data hubs or power extension cables) to suit the needs of the research project. Supplementing the infrastructure may not be possible in other situations because of costs or prohibitive regulations, and researchers may have to settle for less desirable sensors with fewer requirements. Some other examples of the infrastructural considerations that should be accounted for include the following: the total network bandwidth usage for all participants, the availability of power and network outlets, and access to a secured network for sensitive or private data transfer.

#### Criteria Related to the Evaluation of Sensor Characteristics

The criteria in this section describe various ways to evaluate sensors compared with other potential sensor choices. Each choice poses a certain set of constraints on the study, which can affect the research team, the study objectives, and the participants; thus, this merits vigilant consideration.

##### Sensor Customizability

These criteria address the alterability of sensor functionality.

###### Hardware Design

Presently, most commercial sensors are not designed with extensibility or hardware-level customization in mind. Therefore, it is difficult to alter the sampling rate, storage capacity, or battery life to suit the needs of a research study. There exist customizable do-it-yourself (DIY) hardware platforms (eg, Arduinos or Raspberry Pis) that researchers may want to consider in cases where no existing ready-made option is sufficient.

###### Software Customization

Many sensors on the market, which stream data to a smartphone, have a companion phone app, typically providing data visualization, high-level data summaries, or some types of behavioral interventions (eg, *stand up and stretch*, or *get extra sleep tonight*). Some devices, such as smartwatches, contain their own displays for visualizing data and haptic feedback for alerts and interventions. These features can be useful to participants, but they may misalign or interfere with the goals of a research study; therefore, customized versions may be desired.

Certain sensors offer software development kits, enabling researchers to build their own software for collecting, visualizing, and storing sensor data. Other devices, such as the Apple Watch or Wear OS–enabled gear, support software extensions installed on the device, giving researchers more control over the visual and haptic feedback to suit the needs of a study.

##### Cost

The total monetary cost of a sensor device itself is an important factor for researchers to consider, and it may impact the total number of participants who can be recruited and supported throughout a study. Sensor prices can sometimes be negotiated with their providers, depending on the number of devices desired.

##### Battery Life

Sensor battery lives vary greatly and depend on the device types and their functionality. On the basis of the survey of devices available today, wearable sensor battery life spans range from several hours to nearly a week. Most devices are rechargeable in just a few hours, but researchers should offer suggestions to participants about when to recharge to maximize the analytical utility of the data. Some strategies for minimizing the impact of data loss caused by recharging are as follows: staggering the recharge periods for different participants (so at least some data are always present) and choosing recharge times that coincide with periods where the devices could not normally be worn anyway (eg, while sleeping or taking a shower). It is inevitable that participants will at times forget to recharge their sensors, and researchers should have a plan for handling these situations as well. Other devices, such as many tiny and portable environmental sensors, consume a small amount of power, and they can operate continuously for over a year. These devices are often not designed for recharging, and they may need to be replaced throughout the research study.

##### Operating System Support

Some wearable sensors designed to stream data to a smartphone companion app may only support phones running on a particular operating system (eg, iOS or Android), which can create difficulties for the research team. Researchers could elect to recruit only those participants with compatible smartphones, but this will introduce a selection bias that may impact the generalizability of the research findings or may reduce the number of potential participants. If researchers determine that a sensor with partial smartphone support is necessary, these negative effects could be mitigated by providing the interested participants using incompatible smartphones with a temporary and inexpensive compatible smartphone for use during the study.

##### Robustness

These criteria concern the ability of sensors to endure repeated use and proneness to failure.

###### Physical Design

Different sensors have distinct physical characteristics that make them more or less suitable for reliable operations over an extended period of time. Some properties worth evaluating are as follows: whether a device is sturdy and can handle mild physical wear, how easily a worn device may fall off, whether its buttons and other inputs function well after prolonged use, how well it stays in place without shifting, and how quickly it resumes operation after being reattached. Researchers should consider performing a pilot study to fully understand and evaluate the sensors beforehand.

###### Firmware

The reliability of a sensor's firmware is important, as any failure may lead to loss of data. A few probing questions worth answering are as follows: is the firmware code stable or does it crash? Can it handle a barrage of unexpected inputs and continue to function? If the device sleeps, does it resume data collection once awakened? Researchers should stress-test sensor firmware before committing to any device to ensure they understand the possible failure modes and recovery procedures.

###### Companion Software

Some sensors require a companion app running on a second device, such as a smartphone or computer, to facilitate data collection and long-term storage. This software needs to be resilient to minimize data loss. Ideally, it functions consistently, showing no signs of glitches or crashing. Its ability to receive data from the sensor and either cache or upload data to a data server should be seamless and fault tolerant. Research teams should stress-test this software to understand when and how it fails, so that the support staff will be prepared to help participants. A few tests worth performing are as follows: disconnecting the sensor from the app or removing the network uplink during a data transfer to see if sensor data are lost and switching foreground apps or providing random inputs to see if the app crashes. Once the failure modes of the companion software are understood, steps can be taken to remedy them or at least to alert participants.

##### Provider Support

Some companies are interested in building a scientific reputation for their sensor products; therefore, they are concerned with supporting research studies. This support comes in a few forms, and the criteria below pertain to the beneficial impact this support can have during and on the outcome of data collection.

###### Prestudy Support

Before data collection, it is essential for researchers to fully understand the properties and unique characteristics of each sensor under consideration to make the most informed choices. Virtually all sensor providers offer documentation and a communication channel for answering specific technical questions. Some of these providers may offer additional services for research teams, including direct communication to key technical or support personnel and free samples for testing.

###### Logistics

Research teams should seek any available logistical aid, offered by the sensor product companies, that may help the study function more smoothly. Teams should ensure that sensors can be provided on time and that there is a backup plan for any sensors that need replacement. It is advisable to seek help from the product companies to train the research staff for proper fitting of the sensors, especially for those requiring specialized knowledge. Other kinds of logistical help may include preconfiguration of sensors (eg, to specific Wi-Fi networks), custom delivery options (packaging, rush shipping), tailored fittings, or an emergency contact. Moreover, some sensor providers offer ongoing assistance, ranging from providing quality metrics and statistical reports of the study participants to ensuring APIs support the types of data monitoring and quality assessment metrics researchers desire.

#### Criteria Related to Participant Engagement

These criteria pertain to how sensors affect the participants’ perception of a research experiment and willingness to engage with a study throughout its duration.

##### Cohort and Individual Suitability

These criteria relate to the ability of sensors to meet the needs of members of a cohort.

###### Sizing and Fit

Garments and sensors that match each participant’s unique physical characteristics are best equipped to provide usable data. Devices that are too large or too small can cause discomfort, possibly leading to side effects, such as blistering or reductions in data quality.

###### Technological Literacy

Each sensor provides a unique interface for operating with its hardware and companion app software. Researchers should ensure interfaces are simple enough for all potential participants in the target population. In cases where the interface is unfamiliar, researchers will need to provide instructions, describing not only how to operate and interact with the devices but also how to check that they are in a proper state and performing the desired function at any time.

###### Fashionability

The selected suit of sensors should comply with dress codes of the environment in which they will be worn. Moreover, the design and appeal to wear sensors should be considered by the research team to ensure that all participants are comfortable wearing the sensors from an esthetic perspective.

##### Burden to Participants

These criteria address the physical and mental burdens sensors impose on research participants.

###### Physical Interference

Obtrusive sensors may physically interfere with normal activities, causing frustration or eventually leading participants to avoid wearing these sensors or drop out of the study. For example, undergarment or chest strap sensors may become uncomfortable after a few hours or produce skin rashes, preventing participants from using them further. Another example is desk-mounted, infrared eye-tracking devices that require participants to keep their heads in view, which may incidentally encourage poor posture. Other job-specific scenarios should be considered, such as the use of smart rings in hospital settings, where they can interfere with minimal hygiene requirements. Sensors that can adequately collect the intended signals without interfering or causing discomfort will improve the participants’ acceptance of the devices, potentially minimizing attrition.

###### Time Investment

Studies conducted in the wild, which ask participants to wear or interact with sensors over an extended period, inherently push more responsibility onto the participants to manage and operate the sensors. Daily upkeep, such as cleaning and charging the devices and verifying that they are functioning as intended, requires a time investment that burdens participants and can cause frustration if the demands are too high. Choosing sensors with low upkeep and training costs will reduce this burden and can improve compliance and overall data quality [37].

###### Cognitive Load

An implicit stipulation in any study is that the participants understand they are responsible for adhering to the study protocol. This requires that participants remain mindful of the study throughout its duration. Researchers should aim to choose sensors and an overall study design that requires a small or occasional investment of the participants’ time and mental energy. For example, helping participants with reminders to charge their sensors every night, and supporting them with a charging hub may increase sensor usage.

#### Criteria Related to Protection of Human Subjects

Research investigating human behavior, using sensing technology, is subject to review by IRBs, which evaluate the risks and benefits to human participants and ensure that the study adheres to ethical principles detailed in the Belmont Report [38]. Researchers must consider how the passive collection of behavioral data will respect participants’ autonomy and privacy, how it will maximize the benefits of the research while minimizing risks to participants, and how it will ensure that benefits and risks are equitably distributed. Some of the most relevant themes are reviewed here, but it is important to be aware of ethical guidelines that apply to specific populations or data types. Connected and Open Research Ethics CORE provides a checklist to guide researchers in deciding which technologies are appropriate for a study, with respect to protecting human subjects [39].

##### Access and Usability

Researchers are responsible for ensuring that potential benefits of a study are likely to apply to all members of the population under investigation. This means that sensor selection must not inadvertently exclude members of the study population from participation or result in poorer data quality because of individual differences. For example, wearable sensors may be affected by factors related to body shape and size, skin tone, body hair, or tattoos. It would violate the ethical principle of justice to exclude individuals as study participants on the basis of these factors, solely as the sensors selected did not perform well on them. Researchers should aim to select sensors that have demonstrated validity across diverse participants (eg, a heart rate monitor that relies on ECG instead of optical technologies), can be adapted to individual differences (eg, a respiration monitor that can be worn on a bra or belt), and employ inclusive design features (eg, accessibility settings to accommodate those with visual impairments) to ensure equitable representation and data quality.

##### Privacy

Privacy refers to the persons’ right to control what information about them is shared, with whom it is shared, and how these data are used. The most common privacy protection is to separate information that could identify the participant from the data collected about the participant, but some passively collected behavioral data are inherently identifiable and sensitive. For example, GPS features can predict depression symptom severity [40], and 95% of individuals can be identified with as few as 4 GPS data points [41]. Participants electing to engage in a study that requires the collection of sensitive and personal data need assurances that researchers will take steps to mitigate the risk that their behaviors can be linked to their identities.

Given the array of data types available through passive sensing technologies and the low cost of collecting data unobtrusively, it is tempting to collect as much data as possible. However, researchers are ethically obligated to only collect data that are pertinent to specific research questions. When possible, researchers should disable sensors that are irrelevant and securely dispose of data that are not specifically related to study aims. In addition, participants should be able to select which data they are willing to share, with whom, for what duration, and for what purpose. Ideally, sensors should allow participants to deny or revoke access to particular data types. If these user controls are not permitted by third-party providers, researchers should consider providing additional data management tools that help participants exercise their right to privacy.

Many sensors on the market today require participants to register their own accounts, using their own personal information, which creates a link between potentially sensitive data and each identifiable participant. Studies needing to access these data while guaranteeing participant privacy have a few options. Researchers could register dummy accounts, allowing the participants to remain anonymous, or they may alternatively acquire data directly from each participant’s personal profile (eg, by using an API) and then immediately remove PII. In the latter case, researchers should also check that both the network channels from the sensors to data servers and the network channel for researchers to access the data are encrypted and secured to avoid any privacy breaches.

##### Data Security

Proper protection of the PII sensor data gathered from participants requires all communication channels for the data streams to be secured (refer to Figure 2), and it requires protected long-term data storage with limited accessibility. Information sent over a Bluetooth link is naturally secure, as only paired Bluetooth devices can communicate. Similarly, USB transfers are secured between the 2 devices at either end of the USB cable. Data sent over Ethernet or Wi-Fi require an extra encryption layer (eg, https, secure file transfer protocol) to ensure the information cannot be intercepted. RFID and near-field communications are generally not considered safe, but sensors are typically using these channels to infer information (eg, about the movement of people indoors) rather than transferring PII directly. Stored data are typically secured by limiting physical access to the storage device itself, but encryption of the data is also possible. Access to the sensitive stored data should be limited to select members of the research team, and it is usually controlled through credential-based authentication (eg, usernames and passwords). Unfortunately, today, there are many other ways for hackers to obtain PII data (eg, malware, spyware, and cyberattacks), and research teams and participants may wish for every precaution to be implemented. Readers are referred to the study by Filkins et al [42] for more information about protecting private data in a mobile sensing landscape.

### Current Sensing Technology

Much research has remarked on the variety of options and capabilities of sensors for research purposes [43-47], including several tests [48-50] and validation experiments [31,51-54]. These studies overlook the qualities of each sensor, which make them more or less suitable for different research applications.

**Figure 3:**
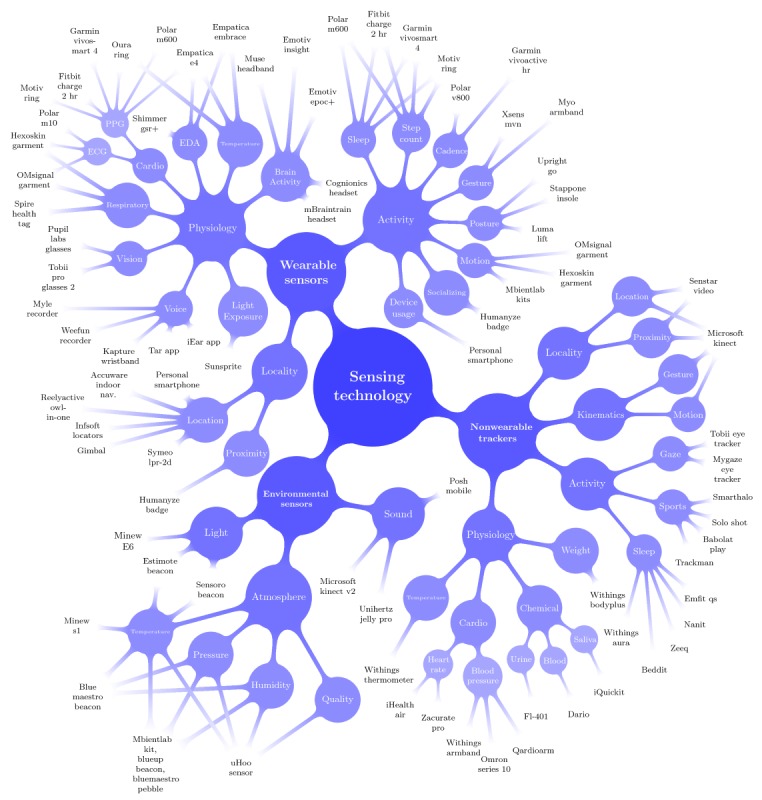
A snapshot of current consumer and research sensing technologies for human behavior studies in natural environments. This is not an exhaustive diagram of sensors on the market, but it provides an overview of the kinds of data can be captured using readily available technology. PPG: photoplethysmography; ECG : electrocardiography; EDA: electrodermal activity.

This section provides a snapshot of some of the more recent and prominent unobtrusive sensing technologies worth considering for human behavior studies in noncontrolled environments. Figure 3 provides a visual overview of this landscape. The following subsections give basic descriptions of these sensors, along with comments about the research trade-offs among different technologies. Compiled in mid-2018 while preparing a research effort examining the relationship between human behavior patterns at home and work, as well as mental states and job performance, the snapshot provided here covers a wide range of sensors that can capture many different signals. The current pace of innovation in the sensing market is too rapid to summarize in its entirety. Therefore, although this list is not exhaustive, it provides an approximate overview of the currently available products. The authors hope these suggestions will be beneficial to researchers formulating large-scale studies.

To make this compilation easier to understand, the sensing technologies are categorized with respect to the type of information they capture. At the highest level, the sensors are grouped into 3 categories: environmental sensors, nonwearable trackers, and wearable sensors, which are also reflected in Figure 3. *Environmental sensors* encompass the devices that capture information about the surrounding environment, which are not intended to directly measure information about people. *Nonwearable trackers* describe devices that are placed in an environment and capture information about people and their behaviors. *Wearable sensors* are the portable devices worn or carried by people, which capture physiological or behavioral data.

#### Environmental Sensors

As the *Internet of Things* movement continues to push more technology into portable devices, environmental sensors that capture multiple kinds of data from their ambient surroundings have become common. Many of the devices in this category are small, battery powered, and can easily be stuck onto walls or placed out of the way. Although their primary purpose is to collect and report environmental data, these sensors can also be used to capture other kinds of information. For example, protocols have been developed for proximity awareness and location-based services, including iBeacon, Eddystone, and Quuppa.

This section focuses on the environmental sensing capabilities of these multipurpose sensors, particularly on measurements of light, sound, and atmosphere. Figure 3 shows a breakdown of these properties and lists several products available on the market, which can be used for measuring each type of data. Environmental sensors are typically designed to remain turned on and collect information from their surroundings at a fixed rate. This information is usually made available to surrounding data hubs or smartphone devices so it can be stored or monitored by people (see Figure 2).

##### Atmosphere and Light

Many of the environmental sensors listed in Figure 3 use microelectromechanical systems devices to measure various properties of the environment. This technology has enabled sensors to be miniaturized and deployed with year-long battery lives, but this comes with a small cost in measurement accuracy. The underlying chips used to assess light levels [55], air pressure [56], and air quality [57] have very low measurement errors; therefore, any inaccuracies will likely be imperceptible at a human scale. The chips often used to measure temperature and humidity have standard errors that may be more significant: approximately 0.5°C and 3% relative humidity, respectively [58]. Near the boundaries of acceptable temperature ranges for controlled indoor environments [59], an error of 1°C may represent the difference between someone feeling comfortable or not. Having this kind of measurement error means researchers would not be able to distinguish between these 2 states. Deploying duplicate sensors in the same environment provides some redundancy in measurement, which allows these errors to be averaged out and may offer more discriminative power during analysis.

##### Sound

Portable sensor products for measuring or classifying ambient sounds are not widely available on the market. The Microsoft Kinect camera is a device capable of capturing audio (and video), but it requires a connected computer, operates on wall power, and is not easily portable. Researchers interested in capturing audio with portable devices in an unobtrusive manner have a few options. The Electronically Activated Recorder [60] periodically samples ambient audio from a smartphone in short bursts throughout the day. Several DIY platforms are also purchasable, such as the Raspberry Pi and Arduino, which can be customized to record microphone audio and transmit acoustic information via Wi-Fi or Bluetooth. Audio signal quality is highly dependent on the setup of the microphone [61] and the types of sounds that researchers want to capture or filter out. The DIY platforms typically offer small omnidirectional microphones with midrange quality, but higher-grade microphones can be integrated. If human-produced audio is of primary interest, researchers may consider a similar solution, such as TILES Audio Recorder (TAR) [27], which is described further in the *Wearable Sensors* section.

## Nonwearable Trackers

Nonwearable trackers live in the environment and monitor and capture information about human behavior. These sensors can be categorized according to the types of signals they are primarily designed to capture: activity, kinematics, locality, or physiology. Some of these devices are self-contained and offer both a data collection mechanism and a means to transmit the information to a data server without a data hub. As these types of devices are not constrained by portability, they can sample data at a higher rate and may also offer more functionality. This section concentrates on nonwearable trackers designed for use at home or in an office.

### Activity

Mentioned below are some of the activities that consumer nonwearable devices can track.

#### Sleep

Sleep monitoring sensors are installed in the bedroom, and these can capture information about the ambient environment and personal physiological information of someone while resting. These sensors may be installed under the mattress, on a nearby nightstand, or on a wall near the bed. They can collect rich information about sleep patterns, tossing and turning, sleep cycles and duration, snoring, temperature, humidity, breathing, and heart rate, and they can ultimately provide details that can be used to assess the quality of sleep.

#### Sport Activities

Many unique kinds of nonwearable sensors used to track sport activities are available, including radars, cameras, tablet apps, and sensors built into sport equipment itself. Some sensors come integrated into sports gear, such as bikes, balls, helmets, and even tennis rackets, each of which blurs the line between wearables and nonwearable trackers. These devices record data, such as posture, gesture type, gesture accuracy (eg, golf swing), and practice duration, and these devices offer approximations for exercise intensity and calories burned.

### Kinematics

Mentioned below are some kinematic features of human movement that can be captured by consumer sensors.

#### Motion

Human body movement dynamics have been linked to affect and cognition [62]. Camera-based computer vision systems allow noninvasive, scalable, and inexpensive motion tracking. A critical issue with camera-based tracking systems is the big brother effect (see *The Big Brother Effect* section). To mitigate participant concerns, researchers may extract relevant kinematic features from the videos in real time and store only relevant features, instead of the video recordings themselves. If physical space is available and the budget permits, using multiple cameras can improve tracking accuracy. Among the available off-the-shelf devices, the Microsoft Kinect [63], particularly with its depth camera technology, is a common choice for real-time full-body motion capture and gesture recognition. Motion tracking of multiple people in a fixed space is possible using cameras, with solutions such as CrowdVision.

#### Gesture

The camera-based systems described in the *Motion* section can also be used to track gestures, especially the Microsoft Kinect [63]. There are freely available tools, such as the Gesture Recognition Toolkit [64], that enable gesture recognition from any video source. Gesture recognition for multiple bodies in real time from single-camera sources is an active area of research, and there are no consumer products available.

### Locality

Descriptions of sensors that capture rough estimations of position (proximity) and more precise location measurements are provided below.

#### Proximity

Modern personal smartphones support Bluetooth and can be programmed to broadcast Bluetooth packets at fixed rates for proximity sensing [65]. Proximity is measured using Bluetooth hubs that receive these broadcasts and use the received signal strength indicator values to determine a smartphone’s approximate distance from each hub. This information can be used, for example, to monitor the amount of time participants spend in front of their workstations or in break rooms.

#### Location

Participant location data are a valuable information channel for human behavior monitoring, offering a means to track the movement patterns of individuals. Like proximity sampling and kinematics tracking, camera-based systems have been widely used for localization in indoor settings [66]. These camera-based solutions require the positions and orientation of each camera to be known. Usually, the cameras are placed out of the way and remain stationary, but they may require maintenance if perturbed. RFID systems are also available on the market, and they use distributed hubs to localize individuals wearing RFID badges. Usually, the location of individuals can be inferred more accurately when more cameras or hubs are added to the environment. It is recommended that research teams test localization systems in the target environment before committing to a solution.

### Physiology

As shown in Figure 3, nonwearable sensors can measure a wide variety of physiological signals, including weight, body temperature, chemical indicators, and cardiovascular information.

#### Weight and Body Mass Index

Some smart scales can measure weight (mass) and assess a subject’s body mass index (BMI) by using bioelectrical impedance through the bare feet [67]. Devices, such as the Withings Body+, capture both of these measurements [68], and they can also assess a person’s total body water (TBW) [69]. Weight and BMI values are linked to obesity risk and heart disease [70], and TBW is linked to subjects’ hydration levels [71]. These signals may be of interest to certain research studies, and presently, these signals are difficult to measure using wearable sensors.

#### Body Temperature

Body temperature is a known proxy measure for health and arousal [72]. Infrared thermography devices [73] measure skin surface temperature without direct body contact. These types of sensors are fast and easy to operate, but the accuracy and precision may be worse for some individuals [74]. Researchers desiring to use these types of no-contact body thermal sensors are encouraged to average several measurements to improve data quality.

#### Chemical Indicators

Nonwearable smart sensors are available, and they can sample blood, urine, or saliva to measure blood glucose level, blood oxygen level, and pH level. Measurements from these body fluids provide rich information about health and well-being, and they are considered the gold-standard source for some signals. These devices may require additional consumables for measurement (eg, electrochemical strips).

#### Cardiovascular

A variety of available nonwearable sensors provide cardiovascular information, such as blood pressure and heart rate. For example, pulse oximeters collect heart rate and blood oxygen saturation from pulse waves measured via PPG at the fingertips. Pulse oximeters are widely used, given their ease of use and low cost. Smart blood pressure sensors are also available, such as the Withings or Omron 10 Series blood pressure monitors. Although these sensors must be attached to the body to capture cardiovascular data, they are not intended to be worn for any length of time. Recently, noncontact, video-based methods of inferring heart rate have been proposed and shown to obtain accurate and reliable measurements as well [75].

## Wearable Sensors

Wearable sensors are separated into 2 primary groups, 1 for devices that directly capture physiological measurements and 1 for devices that infer behavioral or activity states. A third group encompasses sensors that infer information about the positioning of individuals.

### Physiology

This subsection describes the (relatively unobtrusive) commercial sensors that can capture some of the wide variety of human physiological signals.

#### Electrodermal Activity

EDA, also known as galvanic skin response, can be used to track states of emotional arousal through the skin conductance level and responses. Sensors placed around the fingertips are among the most accurate, but they are more intrusive and likely to interfere with the participants’ daily activities. Wristband sensors, such as the Empatica E4, measure EDA, but they can capture a large amount of noise in the signal when wearers move or flex their arm muscles. In practice, it can be difficult to capture this signal reliably and unobtrusively in the wild, without substantial noise. Denoising these signals to obtain more meaningful measurements is an active area of research [76].

#### Speech

Different wearable devices have been proposed by researchers for understanding emotions and other aspects of speech in social situations, such as the Sociometer [77], the EAR [60] and subsequent iEAR app, and the TAR [27] app. Privacy is a major concern when audio recordings are collected in public settings; thus, some apps, such as TAR, are designed to only collect and record anonymized acoustic features from human-produced audio. Commercial wristband devices for collecting raw audio in the wild are available, such as the Kapture audio wristband or the Weefun voice recorder, but these are designed to capture audio on demand and at the request of the wearer. The EAR and iEAR apps autonomously and periodically record ambient audio, but they may pose a privacy concern, especially for nonparticipants.

#### Vision

Eye trackers allow researchers to study human gaze patterns and points of interest that attract visual attention. Wearable consumer products for tracking gaze typically look like glasses and use cameras to track eye movement relative to each participant’s forward head direction. Some of these products require calibration, where users are asked to look at a fiducial marker to realign the calculated gaze direction. Researchers should be aware that participants may need to calibrate periodically to maximize gaze tracking accuracy.

#### Cardio

Heart rate and heart rate variability measurements have been linked to activity levels, emotional arousal, stress, restfulness, and general fitness [78]. Wearable sensors are well suited to track the heart’s behavior. It is currently possible to obtain unfiltered 200 Hz electrocardiograms throughout the day with chest straps, smart shirts, and undergarments. Wristband sensors offer PPG technology that collects volumetric measurements of blood flow. PPG-based wristbands provide heart rate and, sometimes, blood volume pulse metrics, but researchers often cannot access the unfiltered PPG data, as these are processed and transformed by the hardware in the device (usually to save power). Presently, heart rate information, as provided by PPG sensors, may not be accurate (see *Data Properties and Quality*), as evidenced by the study by Benedetto et al [48]; therefore, researchers should exercise caution when using this technology.

#### Respiration

Many chest strap [79] devices that capture ECG data are also capable of measuring respiratory information. These devices use stretch sensors in the strap wrapped around the chest to capture inhalation and exhalation and produce breathing frequency and volume per breath measurement. There are also other accelerometry-based devices that attach to the waist and extract the same measures by ignoring all motions, except those caused by breathing. Respiration sensors offer insight into physical activity intensity, recovery, and calmness when participants are at rest [80].

#### Temperature

Skin temperature provides information about participants’ comfort levels, exercise efficiency, and physical well-being (eg, because of a fever). Some wearable garments that already measure ECG and respiration rate (eg, QardioCore) can capture skin temperature conveniently. Some wristband sensors can measure skin temperature as well (see Figure 3).

#### Brain Activity

Devices measuring brain activity using electroencephalography (EEG) have become more abundant in recent years. Numerous portable and wearable EEG headsets exist, with varying numbers of electrodes for capturing voltage levels at the scalp, and each one offers different sampling rates and monitors activity in different regions of the brain (Brodmann areas). Many of these portable headsets transmit data via Bluetooth, enabling smartphone apps to receive, process, and upload the data. Some devices require the data to be received and processed by a companion app running on a personal computer. Although the underlying technology is very similar for most devices and a standard exists for electrode placement (the 10-20 system), the captured signals may vary from device to device because of several factors: dry versus wet electrodes, sampling rates, number of channels, and degree of sensitivity to ambient (noisy) electromagnetic radiation.

#### Light Exposure

Sunlight exposure has been linked to sleep behavior and overall mental health. Special-purpose ultraviolet and visible light devices with smartphone integration, such as the Sun-Sprite light tracker, are available and can help researchers monitor participants’ exposure to sunlight during the day. Many of the wearable wristbands that capture heart rate and other physiologic information can also track ambient light levels (see Figure 3).

### Activity Tracking

Unique sensors have been developed to track various physical and contextual human activities, which are outlined here.

#### Socializing

Some wearable technologies can capture information about person-to-person social interactions. Active RFID or Bluetooth devices, such as the Humanyze badge, detect when 2 or more people are standing next to and facing each other. These devices and other wearable voice detectors (previously mentioned in the *Physiology* section) can also help determine when vocal exchanges occur between people nearby. Together, these types of sensors can aid researchers in assessing when group socialization occurs, how long it lasts, and who is involved. In practice, it is often difficult to determine who is speaking, and when anonymized audio features are collected instead of raw audio [81]; deciding whether a vocal utterance is intended as part of a conversation is an open research question. Nevertheless, even noisy inference of group social activity may be beneficial for a research endeavor.

#### Gestures

Human gestures contain valuable information in social contexts and provide insight into kinesiologic activity while at work or at home. Wearable sensors for gesture tracking commonly use IMUs to record motion of the arms and legs, and they may use electromyographic muscle sensors to detect certain kinds of hand gestures. The data can be streamed in real time via Bluetooth to smartphones or data hubs.

#### Motion

Many research efforts have previously observed a strong correlation between physical activity and both physical and mental health. With the explosion of fitness trackers in the last few years, it is now possible to track body movement and exercise patterns with relatively inexpensive wearable devices. These devices come in a variety of form factors with very different qualities and captured signal characteristics. One of the most common forms is the wristband, with other options including chest straps, shirts, and undergarments. Many of these devices contain embedded accelerometers and gyroscopes to record translations and rotations of the body over time. In addition, some devices may directly provide time series IMU data, whereas others may digest this to produce higher-level motion features, such as step count or distance traveled.

#### Posture

Some smart shirts and chest straps (mentioned in the previous section) can also be used to track the posture of participants. These devices contain IMU data that can be processed to obtain information about whether the wearer is sitting or standing, what his or her angle is with respect to the ground, and about sleeping posture (eg, resting on one’s back, front, or side). Other sensors for gesture tracking attach to the arms and legs and can help provide a more holistic view of the entire body’s posture over time.

#### Cadence

Cadence measures capture the consistency of repetition of motion over time and offer information about physical fitness, activity intensity, and physical exertion. Some devices provide cadence measures directly (usually for physical activity), but cadence can also be inferred and analyzed from other available motion modalities, such as IMU data.

#### Sleep

The *Nonwearable Trackers* section mentions nonwearable devices for tracking sleep, but many unobtrusive wearable devices can do so as well. Some wristbands and smart garments that track heart rate and motion can detect when a person is sleeping, and they can infer information about sleep stages and sleep quality from these data streams. Accurate sleep stage tracking from these types of data streams is a continuing area of research, and although many devices offer sleep metrics, they have not been validated thoroughly by the scientific community. These reported values may still be useful, but researchers should be cautious when using these sleep metrics.

#### Step Count

Step count can be inferred from wearables using IMUs that track motion and posture; therefore, it can be measured using wristband and garment devices. Bluetooth-enabled pedometers worn at the waist or tied to shoes are other options. Newer iPhones can also easily measure step count, which is calibrated using GPS information. The number of steps counted for any given activity will vary among devices and across participants because of differences in motion and how the motion is interpreted by the sensors. Particularly, wristband sensors may misinterpret activities involving repetitive arm motion (eg, washing dishes) as steps and introduce measurement error.

#### Personal Device Use

Apps for tracking smartphone usage have been developed to help people monitor and manage their own time, and they can also be used for research purposes. These apps primarily track how frequently users pick up their smartphones and how long they spend using different apps each day. Some of these tools can also track social media and internet use.

### Locality

Some wearable sensors are able to provide coarse approximations of location (proximity) and finer location estimates (localization), which are described below.

#### Proximity

Proximity-based locality measurements yield rough estimations of location by proximity to other *a priori* known locations, usually measures through time-of-flight or received signal strength. The systems available for tracking proximity use a deployment of Bluetooth, Wi-Fi, or RFID hubs, with known locations, to track the presence of wearable devices. Wearable badges and cards can be purchased for each participant, which are detectable by these hubs, but it is also possible in some cases to track devices that participants might already have on their person, such as smartphones. These proximity tracking systems are useful for detecting potential social interactions among nearby people or detecting when people are present in a known area.

#### Localization

Localization is the process of measuring or inferring a precise approximation of a person or object’s location. Most smartphones today provide GPS-based location services, which provide accurate location measurements that are useful for tracking human movement at city scales. Other systems, such as dense Bluetooth or RFID hub network, can be used in conjunction with Bluetooth beacons or RFID cards carried by participants, and they provide precise estimations of their position in indoor environments. Location data are highly sensitive, and extra steps may need to be taken to securely collect, deidentify, and transmit this type of information.

## Results

### Overview

In early 2018, a research team (including the authors) began preparations for an *in situ* study at the University of Southern California’s Keck Hospital, per the MOSAIC program [82], using sensors to track nurse and hospital staff behavior in the workplace and at home. The project aimed to understand how physiological dynamics and behavior both at work and at home are associated with personality, well-being, and work performance.

This section shares results from the application of the methods previously described. The team’s experiences and rationale for selecting sensors to help achieve the research objectives are discussed, as well as how compliance was monitored and encouraged during the study. Metrics for attrition and compliance rates are provided. For a more detailed overview of the data collection itself, including IRB information, readers are referred to the retrospective study by Hasan et al [83]. A full description of the dataset and collection methodology will appear in a future publication; this section focuses on aspects related to sensing and data flow.

### Study Goals and Constraints

The primary goal of MOSAIC was to use information gathered through commercially available sensors to study the predictive power of these types of sensors for assessing personality traits, as well as work-related behaviors and mental states throughout time. Owing to the complex trade space encompassing consumer sensors, creating a data collection protocol that met the project goals and was satisfactory to the participants and hospital environment required many iterations and challenging decisions. These deliberations and the data collection protocol that resulted led to a study, including over 200 hospital staff participants over a 10-week period and with an attrition (dropout) rate of 4% (primarily because of vacation conflicts).

### Signals and Sensors

The signals of interest and sensor selection rationale are described below. The sensors employed in this case study, on the basis of the various study constraints, are also described.

#### Signals of Interest

Previous literature, related studies, and experience all revealed many physiologic signals of interest for capturing data likely related to work behaviors and mental states. Some of these signals, such as EDA and brain waves, were not possible to capture accurately in the wild over extended periods using consumer sensors. The research team initially reduced the list of potential signals of interest down to the ones that could be captured with unobtrusive sensors, based upon a survey of existing technologies (see Figure 3). Table 2 shows these signals and a short explanation of the expected utility for each in meeting the research objectives.

**Table 2:** Signals of interest in the case study that were measurable using consumer sensors.

Signal	Reason for interest
Cardiac	Connection to exercise, fitness level, and stress levels [84,85]
Physical activity	Linked with stress [86]
Sleep	Health (physical and emotional) [87,88]
Speech	Contains information about emotional expressions [89] and information related to social interaction
Breath	Calmness, stress, anxiety, and speech activity detection [80,90]
Environment and distractions	Connection with workplace performance, anxiety, and stress [91]
Locality	Captures workplace behavior and job role dynamics [92] and context for the job types of interest

#### Sensor Selection Rationale

As the study continuously required the collection of data over several months, one of the top priorities was to minimize the burden on participants to achieve a high compliance rate and capture representations of behavior in the wild. As previously described in the *Burden to Participants* section, the study took a holistic approach to assessing the participants’ responsibilities and duties, including their time invested in compliance, physical disruption, cognitive load, and interference with their daily activities. While keeping these burdens in mind, each paragraph below describes how sensors were chosen to capture each signal of interest.

##### Cardiac and Physical Activity

Several form-fitting garments with ECG sensors were tested, and many provided the data quality desired (see Figure 3 for the list). Chest strap sensors were found to be uncomfortable for daylong use (as they are designed for exercise sessions), but the existence of different form factors of ECG garments (eg, shirts, bras) made it possible to gather high-quality data across genders. Some of these garments continuously collected high-quality data throughout the day, but they required that the physical box recording the data and hidden inside was hooked up to a computer via USB on a daily basis for data transfer. This step seemed cumbersome for participants; therefore, another similar garment that could stream the data to the subjects’ personal smartphones was selected. The caveat with this second device was its companion app, which required a manual start and stop of the data recording process. The research team elected to have subjects wear these garments only during work hours to avoid potential discomfort associated with wearing them all day. Participants were also assisted in setting location-based reminders on their personal phones to start and stop the recordings. Heart-related information and other physical activities outside of work were also tracked by asking participants to continuously wear a wristband.

##### Sleep

Many unobtrusive sensors were capable of capturing information about sleep duration and sleep stages. Some sensors required a one-time installation on or near the bed, and then they would automatically detect and monitor participants when the participants were sleeping. Nurse focus groups had privacy concerns; therefore, wearable sensors were deemed more appropriate. To minimize cost and the burden of wearing multiple sensors, a wristband sensor was chosen, which was capable of capturing sleep and the cardiac and physical activity signals mentioned previously. Participants were asked to wear the band every day, including during sleep.

##### Speech

**Figure 4:**
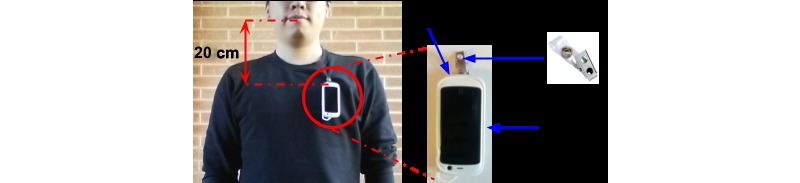
Setup of the TILES audio recorder [27].

At the time of the study, no portable consumer devices were available for automatically sampling only human-produced audio. The research team programmed a smartphone app to automatically start, run in the background, and collect audio samples of ambient human utterances [27]. To address Health Insurance Portability and Accountability Act concerns about hospital patient and nonparticipant privacy, relevant information about the emotional content of the voice signal was computed by the device, and the raw audio signal was immediately discarded. Moreover, participants could disable the recording process for intervals of half an hour, by pressing a button in the app, after which the recording was resumed. Collecting low-noise audio required the smartphone’s microphone to be placed near the mouth, and the research team wished to avoid using external microphones to avoid further participant burdens. Research staff met with representatives from the potential participant pool to discuss unobtrusive solutions and discovered that hospital personnel were already accustomed to wearing hospital badges on their lapels. Credit card–sized smartphones were acquired to run the custom software, and then these were attached to the participants’ shirts, with a clip to get the microphone closer to the mouth [27], as shown in Figure 4. Although this solution may have been unacceptable for some subject populations, it was appropriate for the hospital workers in this study.

##### Breath

Commercially available portable breath sensors measured the expansions and contractions of the chest. Some of these sensors were stand-alone devices attached to the waist or chest, and some were integrated into other multipurpose sensing garments. Once the research team decided on a comfortable device for capturing ECG, they found that breathing rate information was already available; therefore, the same device was used.

##### Environment and Distractions

Environmental sensors for capturing temperature, humidity, and door motion were used. Statistics about social media and general phone usage were acquired with the participants’ permission and with the help of smartphone apps running in the background on their personal phones, requiring little power and no interaction after the initial setup.

##### Locality

Precise localization of subjects inside the hospital was deemed prohibitively expensive and would have required several months of installation time; therefore, approximate measurements of location by proximity to known locations were used instead. As described previously in the *Wearable Sensors* section, using a dense hub network and wearable consumer sensors, there were 2 general ways to achieve this: tracking participants’ smartphones or tracking other worn wireless communication devices. The latter option was chosen using the audio recording phones for tracking to avoid any power draw from participants’ personal phones.

#### Selected Sensors and Expected Use

Table 3 shows the selected sensors and the intended usage period for participants, per the study protocol.

**Table 3:** Selected sensors and their expected use.

Sensor	Measurements	Intended usage period
Fitbit Charge 2	Photoplethysmography-based heart rate, step count, and sleep	24 hours per day
OMsignal garments	Electrocardiography-based heartbeat, breath, motion	At work (12-hour shifts)
Unihertz Jelly Pro	Audio features, Bluetooth-based localization	At work (12-hour shifts)
reelyActive’s Owl-in-One	Bluetooth-based localization, data hub for environmental sensors	Installed at the University of Southern California’s Keck Hospital, 24 hours per day
Minew E6, E8, S1	Light, motion, temperature, and humidity	Installed at the University of Southern California’s Keck Hospital, 24 hours per day

### Data Flow

Figure 2 depicts a general flow of information for measurements obtained through sensors. In the study, all 3 kinds of sensors (in the left column) were used: environmental sensors, nonwearable, and wearable. All of the sampled data flowed through 2 different intermediate types of data hubs: Bluetooth data hubs connected to Wi-Fi and personal smartphones. Personal computers were not used to retrieve any data in an effort to reduce the time spent by participants uploading data to different servers.

Wireless passive sensors capturing information about light levels, temperature, and humidity were used, which transmitted information over Bluetooth. In addition, the participants wore Jelly Pro phones that were programmed to send Bluetooth pings with unique identifiers. The Owl-in-Ones received the data. They were connected to the public Wi-Fi network of the hospital and transmitted the data over this network to reelyActive’s servers, from which the data were retrieved in real time, using a provided API.

Audio data recorded by Jelly Pro phones were directly uploaded to the research server, using hospital or home Wi-Fi networks. Wi-Fi was necessary because of the size of the files, approximately 8 GB per day.

Data transfer took place from Fitbit Charge 2 devices to participants’ smartphones over Bluetooth, followed by data upload to Fitbit’s servers through the smartphones’ internet connections. The research server then retrieved these data using Fitbit’s API. The same flow was employed by the OMsignal garments, using OMsignal’s API.

Feedback for participants happened through a custom app (the TILES app) via push notifications. This app sent surveys to participants and gave them notifications about sensor usage and the quality of their previously received data when necessary.

### Monitoring and Encouraging Compliance

Minimizing participant frustration in a study can help improve compliance and overall data quality [14]. This was one of the top priorities in this case study, and this was achieved by reducing cognitive burdens on participants, offering monetary incentives and consistent feedback to participants for compliance and providing convenient help whenever the participants encountered difficulties.

A custom smartphone app for the participants was developed, and it served as the primary resource for all aspects of the study. This app provided progress and monetary reward tracking, information about the study and protocol, and direct contact links for requesting help, and it also distributed questionnaires and reminders. Participants were rewarded for uploading their data daily, per the study protocol, which allowed the research team to monitor compliance and data quality every night. Each morning, the app provided feedback to the participants by letting them know whether their previous day’s data had been received and whether the quality was sufficient. If the data were missing or quality was poor, the app reminded participants to double-check their sensors or seek help from the research team.

On-site assistants were always available during work hours to help participants who encountered difficulties during the study. Participants were able to drop in for help, or they could request for assistants to visit them and provide in-person support. These assistants actively engaged with participants who had recently uploaded poor-quality data to help make sure their devices were worn and functioning properly.

### Metrics

Table 4 shows the average data compliance rates across different 10-week waves of this study for different sensors. The attrition rate was under 4% across all participants, and most of the participants dropped out because of vacation time conflicting with the study’s participant inclusion criteria. More details about the study, including information about poststudy surveys on user experience, are available in the study by Hasan et al [83].

**Figure 5:**
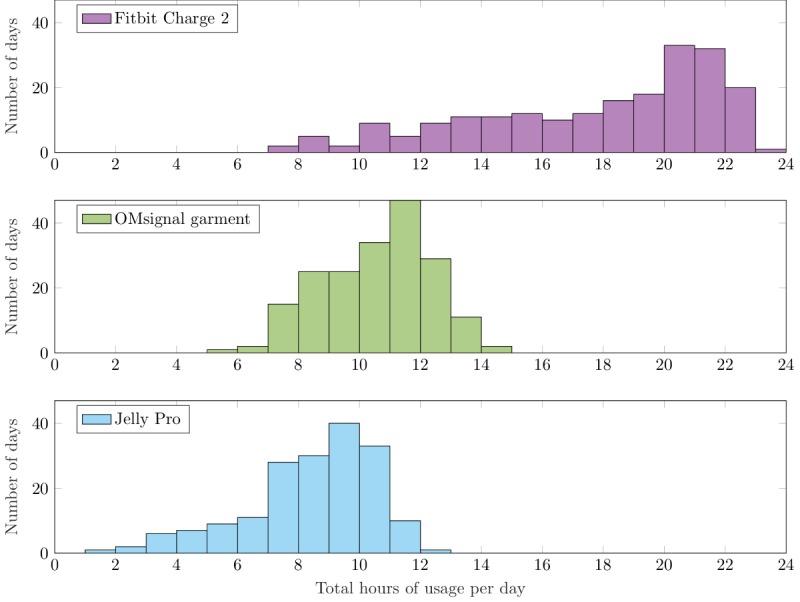
Histograms of the total number of hours of recorded sensor data per day, across all participants. These plots only show data from days where data was logged.

Figure 5 shows a histogram of the number of hours each sensor was used per day across all participants, where days with no logged data are not shown. This figure illustrates that on average, Fitbit was used about twice the amount of time as other sensors, which was in line with expectations. Moreover, although both the Jelly Pro and OM garments were designed to be used by participants at work, there is a noticeable difference in usage. This is partly explained by participants starting the recording of their OM garments at home rather than at work. It can also be explained by the fact that the Jelly Pro recording is activated only when participants or nearby persons are speaking.

The Fitbit usage of the subject cohort is in line with other studies [93], which claim 70% to 90% of compliance using wristband sensors. Compliance rates for the OM garments and the Jelly Pro are and were expected to be lower, as these devices required more attention from participants.

For Fitbit, the mean usage among all days with logged data is 17.8 hours, with an SD of 4.0 hours. For the OM garments, the mean is 10.6 hours, with an SD of 1.8, and for the Jelly Pro audio recorder and localizer, the mean is 8.4, with an SD of 2.1 hours.

**Table 4:** Compliance rates for participant-tracking sensors (n=212) and environment sensors (n=244) in the case study.

Sensor type and signals		Sensors	Participant who opted, n (%)	Total hours	Compliance rate^a^, n (%)	Definition of compliance
**Participant-tracking**						
	Cardio, sleep, and steps	Fitbit	208 (98.1)	236,725	152 (73.1)	Average fraction of days per participant with >12 hours of data
	Cardio, breath, and motion	OMsignal	208 (98.1)	44,240	125 (60.1)	Average fraction of work days per participant with >6 hours of data
	Audio	Jelly	184 (86.8)	37,065	131 (61.8)	Average fraction of work days per participant with >6 hours of data
	Locality	Jelly+Owl-in-one	184 (86.8)	37,065	131 (61.8)	Average fraction of work days per participant with >6 hours of data
**Environment**						
	Temperature, humidity, and motion	Minews	—	—	239 (98.0)	Uptime of the sensor network

^a^Compliance is computed as the presence of data exceeding half of the measurement period per day among the participants who opted in for each sensor.

## Discussion

### Principal Findings

The methods previously presented are evaluated with respect to the case study outcomes in 2 ways: participant compliance and the number of emergent unexpected challenges during the data collection period. More details are provided in the following subsections, but they can be summed up in the following manner: (1) participant compliance was satisfactory, yielding a large average number of hours of data per participant per day; (2) the unexpected challenges were manageable and had either very short-lived or isolated impact on the study.

These metrics suggest that the methods and mitigation strategies presented in this paper as a guide for researchers are helpful for sensor selection and management during longitudinal human behavior studies in the wild.

### Participant Compliance

Participant compliance rates in the case study fall within an expected range when compared with observed compliance ratios in similar study conditions from the study by Merilahti et al [93]. The work from the study by Lima et al [94] observes that participant compliance decreases over time in long-term studies, which is also observed in this study. Overall, a sufficient number of hours of data per day per participant are collected for statistical analysis; therefore, the compliance levels are satisfactory.

### Unexpected Challenges During the Case Study

This section recounts the unanticipated challenges encountered during the study despite efforts to avoid them during study planning. Unexpected challenges are defined as the events that were deemed unlikely to happen or that were not considered *a priori*, and these negatively affected the project budget, schedule, participants, or data. Each of the occurrences below were either isolated incidents, affecting only a narrow piece of the research project, or were short-lived, as the research staff was able to address them quickly. The following subsections present potential strategies for mitigating each of these events in future studies.

#### Shipping Dependencies and Customs

Some bundled sensor shipments were delayed because of product dependencies on secondary companies with limited shipping capacities. Urgent sensor package shipments from other countries were sometimes held up by the customs authority. In future studies, it would be best to be aware of the shipping capabilities of each product company and any potential shipping delays when preparing a study schedule.

#### Installation Time

The research staff underestimated the time required to install on-site sensors at the hospital. Although floor plans were used heavily for placement planning, they did not include locations of the electrical outlets. Several iterations and supplemental cabling were needed to install sensors across 16 different nursing units with similar layouts but different electrical circuit restrictions. Moreover, as most sensors were installed in patients’ rooms, more trips to the data collection site were needed than expected to accommodate patient needs. Starting the installation process early can help researchers identify this problem in advance and budget time accordingly.

#### Battery Life

The Jelly Pro devices running the custom TAR app ran out of power for some of the participants early on during data collection. The parameters of the TAR app were tuned on the basis of the data collected during a pilot study from a subset of the final participant pool, but this subset did not reflect the worst-case scenario for power consumption. The battery life in this case depended on how many times vocal audio recording was triggered by the automatic voice activity detector, and the hospital staff in highly social environments triggered it more often than the worst case in the pilot study. The research team responded by recollecting the Jelly devices and modifying the parameters overnight. A possible strategy for mitigating this issue would be to design a pilot study that includes more participants at the expected extremes of the measurement spectra, but this may negatively affect the expected average-case findings. Perhaps a better strategy would be to implement tools to remotely or more easily update the parameters for all participants in anticipation of this type of issue.

#### Sensor Synergy

As the Jelly Pro devices served 2 functions in this study (collecting vocalized audio and proximity detection), when the power consumption exceeded expectations, 2 data streams were affected instead of 1. For sensors serving multiple purposes, there is greater risk to the data quality when they fail; therefore, proper stress testing and tooling (as mentioned in the previous paragraph) should be prepared before the main study.

#### Sensor Discomfort

Some participants acquired rashes caused by skin friction with the wrist-worn or undergarment sensors. This occurred because the sensors these participants used were improperly fitted or sized, and the discomfort they produced led to a short-term loss of data while the participants recovered. The pilot study helped the research staff identify and mitigate some fitting concerns, but it was not enough to handle all the cases during the main study. The team reached out to the product companies for these sensors to get help with proper fitting procedures, and with their guidance, they were able to find proper fits for each affected participant. Better approaches for mitigating the risk of data loss here would be to solicit help with fitting and sizing from the product companies earlier and then incorporate that wisdom into the study (as mentioned in the *Provider Support* section), as well as consider different options for materials that are in contact with the skin (eg, Fitbit offers wristbands of different materials).

#### Data Pipeline Failure

Months into the main data collection, 2 site-wide disconnections of the environmental and proximity sensors occurred. These devices were all connected to the existing hospital Wi-Fi network, and the research server’s data monitoring processes identified this event immediately. Within 24 hours, research staff was dispatched to manually power cycle the devices and ensure they reported gathered data upstream. Although these sensors were stress-tested during the pilot study and determined to be robust to power and network outages, they did not all recover automatically in these 2 instances. Having a separate backup system in place (eg, an extra firmware layer to perform a soft reboot) may help improve robustness in these unexpected situations, but the data monitoring processes enabled researchers to respond quickly in this instance.

### Conclusions

This viewpoint highlights and enumerates many of the research challenges faced during studies conducted in the wild, when using sensors for unobtrusively capturing human activity and behavior; presents a diagram illustrating information flow and an explanation of the roles of different computerized devices for data collection, transmission, and storage; and provides as a comprehensive list of criteria that researchers should carefully consider when conducting their own studies in natural settings, including explanations of trade-offs among them. The paper offers an overview of the state of current consumer technology for unobtrusive sensing in the wild, and it provides a snapshot of many of the products available for measuring different types of environmental, physiological, and behavioral data. The information presented is based on previous work and the team’s experiences in executing a large-scale 10-week study for assessing human behavior, well-being, and performance in a hospital environment using a variety of sensors. The collection of methods and criteria for sensor selection and management were evaluated, using this study, with respect to compliance rates and the impact of unexpected emergent challenges that arose during data collection.

## Appendix

Multimedia Appendix 1 Sensing checklist for studies in the wild.

## Abbreviations

API: application programming interface
BMI: body mass index
DIY: do it yourself
ECG: electrocardiography
EDA: electrodermal activity
EEG: electroencephalography
GPS: Global Positioning System
IARPA: Intelligence Advanced Research Projects Activity
IMU: inertial measurement unit
IRB: institutional review board
ODNI: Office of the Director of National Intelligence
PII: personally identifiable information
PPG: photoplethysmography
RFID: radio-frequency identification
SNR: signal-to-noise ratio
TAR: TILES Audio Recorder
TBW: total body water
USB: Universal Serial Bus
