# Supplementary material for: Multimodal Human and Environmental Sensing for Longitudinal Behavioral Studies in Naturalistic Settings: Framework for Sensor Selection, Deployment, and Management
Source: J Med Internet Res. 2019 Aug 20;21(8):e12832. doi: 10.2196/12832 (PMC6719486; doi:10.2196/12832)
Supplement: Multimedia Appendix 1 [file jmir_v21i8e12832_app1.pdf]

Sensing Checklist for Studies in the Wild

Top Priorities (Mantras)

- Minimize participant burden
- Monitor subject compliance and offer feedback
- Provide assistance for troubleshooting
- Confer with subjects about sensors early

Pre-study Planning (Sensor Survey)

Signals of Interest

- ❏ Sensors capture target signals of interest
- ❏ Expected data quality is high enough for future analysis

Data Properties and Quality (per sensor)

- ❏ Sufficient sampling rate
- ❏ Adequate SNR
- ❏ High accuracy and precision
- ❏ Minimal measurement drift
- ❏ Access to raw data
- ❏ Access to preprocessed data
- ❏ Versatility

Data Access Logistics

- ❏ Easy data upload process
- ❏ Have process for aggregation of data onto research server

Sensor Synergy

- ❏ Redundancy in signal measurement (if desired)
- ❏ Can use one sensor instead of two?

Additional Costs

- ❏ Installation tools
- ❏ Replacement sensors and/or parts
- ❏ Service fees

Sensor Criteria

- ❏ Cost
- ❏ Battery life
- ❏ OS support (Android/iOS, Windows/MacOS)
- ❏ Robustness (physical design, software including firmware, app, server side)
- ❏ Versatility for achieving multiple objectives
- ❏ Provider support
- ❏ Privacy (anonymized data, secure transfer)

Ethics

- ❏ Sensors meet *CORE* criteria
- ❏ Study benefits apply to subject population
- ❏ Data transfer and storage are secure
- ❏ Only relevant data is collected
- ❏ Participants understand how data is used

Participant Engagement

- ❏ Support staff readiness (trained, can help with sizing/fitting)
- ❏ Cohort technical literacy (with training)
- ❏ Participant acceptance of sensors (fashionability, minimal interference)
- ❏ Minimal sensor demands (participant time, attention/cognition)

Pilot Study (Sensor Testing)

Preparations

- ❏ Study site preparation (infrastructural needs, supplemental tools/devices)
- ❏ Sensor data monitoring and reporting (data presence, data quality)
- ❏ Support staff availability

Testing

- ❏ Participant acceptance (data collection protocol, sensor devices)
- ❏ Data monitoring system functions as intended

Main Study (Data Collection)

Maintenance and Support

- ❏ Maintenance plan for sensor failure
- ❏ Monitoring data streams (presence, quality)
- ❏ Providing participant feedback (compliance, incentive progress)
- ❏ Support staff scheduling
